# Stochastic-HD: Leveraging Stochastic Computing on the Hyper-Dimensional Computing Pipeline

**DOI:** 10.3389/fnins.2022.867192

**Published:** 2022-05-30

**Authors:** Justin Morris, Yilun Hao, Saransh Gupta, Behnam Khaleghi, Baris Aksanli, Tajana Rosing

**Affiliations:** ^1^Department of Electrical and Computer Engineering, San Diego State University, San Diego, CA, United States; ^2^Department of Computer Science and Engineering, University of California, San Diego, La Jolla, CA, United States; ^3^IBM Research, San Jose, CA, United States

**Keywords:** Hyper-dimensional computing, stochastic computing, brain inspired cognitive architecture, machine learning, processing in memory

## Abstract

Brain-inspired Hyper-dimensional(HD) computing is a novel and efficient computing paradigm. However, highly parallel architectures such as Processing-in-Memory(PIM) are bottle-necked by reduction operations required such as accumulation. To reduce this bottle-neck of HD computing in PIM, we present Stochastic-HD that combines the simplicity of operations in Stochastic Computing (SC) with the complex task solving capabilities of the latest HD computing algorithms. Stochastic-HD leverages deterministic SC, which enables all of HD operations to be done as highly parallel bitwise operations and removes all reduction operations, thus improving the throughput of PIM. To this end, we propose an in-memory hardware design for Stochastic-HD that exploits its high level of parallelism and robustness to approximation. Our hardware uses in-memory bitwise operations along with associative memory-like operations to enable a fast and energy-efficient implementation. With Stochastic-HD, we were able to reach a comparable accuracy with the Baseline-HD. Furthermore, by proposing an integrated Stochastic-HD retraining approach Stochastic-HD is able to reduce the accuracy loss to just 0.3%. We additionally accelerate the retraining process in our hardware design to create an end-to-end accelerator for Stochastic-HD. Finally, we also add support for HD Clustering to Stochastic-HD, which is the first to map the HD Clustering operations to the stochastic domain. As compared to the best PIM design for HD, Stochastic-HD is also 4.4% more accurate and 43.1× more energy-efficient.

## 1. Introduction

Brain-inspired Hyper-dimensional (HD) computing has been proposed as a light-weight computing method to perform cognitive tasks on devices with limited resources (Kanerva, 2009; Imani et al., 2017a) for cognitive tasks such as activity recognition, object recognition, language recognition, and bio-signal classification (Rasanen and Saarinen, 2015; Rahimi et al., 2016b; Imani et al., 2017b). HD computing works by emulating the sparse distributed memory used in the brain that stores information in spare high dimensional vectors. HD computing has three main stages, (1) Encoding: mapping data into *hypervectors* (HVs). (2) Training: combining encoded HVs to create a model representing each class with a HV. (3) Inference: comparing the incoming sample with the trained model to find the most similar class.

HD computing has highly parallelizable operations by operating on independent HVs with 10, 000 dimensions. However, during encoding and inference, there are numerous element-wise multiplies and subsequent accumulations due to matrix multiplication(dimension 10, 000) which, when mapped to processing-in-memory (PIM) architecture, have to be performed sequentially.

Table 1 shows the breakdown of inference and the percentage of time spent working in parallel or sequentially due to these operations. The XOR operations are able to work completely in parallel, while the accumulation (which includes the element-wise multiplication) needs to work sequentially as the operations are not simple bit operations.

**Table 1:** Execution time breakdown of a naive-PIM implementation (Imani et al., 2019f).

**Similarity check** **breakdown**	**XOR**	**Accumulation**	**Accumulation (D × larger memory)**
Naive PIM (Imani et al., 2019f)	3.3 ns	13,504 ns	97 ns

These operations cause a clear bottleneck for HD computing in PIM architecture. Although this can be alleviated by significantly increasing the memory size, that comes at a *D*× increase in area, where *D* = 4, 000. Prior work leveraged simple analog PIM memory to implement HD Computing (Imani et al., 2019g) to solve this issue but they lose, on average, 3% of the accuracy of HD Computing because the analog circuits are too aggressive at approximation. To alleviate this issue, we utilize Stochastic Computing (SC) operations,a computing paradigm that uses random bitstreams to convert complex computations to simple bit-wise operations on the streams (Gaines, 1969), in their place, which are much more hardware friendly for PIM architectures. Additionally, with SC, we are able to maintain the accuracy of HD better than previous aggressive analog PIM architectures.

In this paper, we leverage Stochastic Computing (SC) during the encoding, training, and inference phase of HD computing to create an end-to-end stochastic implementation of HD. We propose, Stochastic-HD, which combines both HD computing and SC to perform classification tasks in PIM with highly parallel operations. To do this, we utilize deterministic SC (Jenson and Riedel, 2016), which uses a more structured way instead of typical randomly generated bitstreams to represent the bitstreams thus results in better accuracy. Stochastic computing enables our design to remove previous reduction operation bottle-necks for a more efficient and fast design. By integrating retraining in the stochastic domain and creating an end-to-end implementation, this version of Stochastic-HD, is able to reach a comparable accuracy with the Baseline-HD losing only 0.3% in accuracy on average. Furthermore, this work is the first to extend Stochastic-HD to support HD Clustering. We additionally, extend our hardware accelerator to support retraining, as well as HD Clustering in Stochastic-HD creating an end-to-end accelerator for Stochastic-HD Classification and Clustering. As compared to the best PIM design for HD (Imani et al., 2019g), Stochastic-HD is also 4.4% more accurate and 43.1 × more energy-efficient.

## 2. Related Work

### 2.1. Hyper-dimensional Computing

Prior work applied the idea of HD Computing to different classification problems such as language recognition, speech recognition, face detection, EMG gesture detection, human-computer interaction, and sensor fusion prediction (Rasanen and Saarinen, 2015; Rahimi et al., 2016a,b; Imani et al., 2018, 2019d; Kim et al., 2018). Prior works also proposed binary encoding to accelerate HD Computing (Imani et al., 2019a,b,e,g; Morris et al., 2019).

Prior work has also proposed hardware accelerators for HD Computing such as FPGAs (Salamat et al., 2019), and PIM architectures (Gupta et al., 2018; Imani et al., 2019g). Although GPUs and FPGAs provide a suitable degree of parallelism that makes them amenable to machine learning algorithms such as deep neural networks, the complexity of their resources, e.g., floating point units or DSP blocks, is by far beyond HD's requirements, making such devices inefficient for HD. PIM architectures tackle this problem as they are comprised highly parallel arrays with intrinsically non-complex computational capability, which is sufficient for HD operations. Additionally, PIM can eliminate the high cost data movement in the traditional von Neumann architectures as, in PIM, data resides where computation is performed. Adding a PIM accelerator for HD computing to perform cognitive tasks provides significant speed up over utilizing the on-board CPU and saves energy with less data movement.

Previous work also mapped HD computing to deterministic stochastic computing (Hao et al., 2021). However, this design only supported HD Classification encoding and similarity check to perform inference. The key piece missing from prior work is integrated retraining and supporting other machine learning applications such as clustering. Without integrated retraining and simply converting hypervectors to the stochastic domain, there is a significant accuracy loss of 1.3% on average. In this work, we extend the capabilities of this design to support retraining. This closes the accuracy loss gap to just 0.3% compared to exact operations on average. Furthermore, to the best of our knowledge this is the first work to propose HD Clustering in the stochastic domain.

### 2.2. Stochastic Computing

Prior work has utilized SC (Gaines, 1969; Qian et al., 2010; Alaghi and Hayes, 2013) as a low-cost computing paradigm which process random bitstreams for implementations of convolutional neural networks (Kim et al., 2016; Ardakani et al., 2017; Lee et al., 2017). To calculate *p*×*q* for *p, q*∈[0, 1], SC will generates two random independent binary bitstreams, where the probability of a “1” in the first and second bitstream is p and q. For example, the stream 1010011110 could represent 0.6 since it consists of six 1's with a total of ten bits. With converting numbers into bitstreams and using a single AND operation to perform multiplication, SC successfully reduced the power and cost of complex but necessary operations such as multiplication in conventional implementation of neural networks. The usage of long bitstreams to represent data also ensures that SC implementations are noise tolerant.

However, as shown below, when the bitstream length is not long enough, with random arrangements of 1's and 0's, the result of ANDing two bitstreams may not be exact. The random fluctuations resulted from the generation of bitsteams cause the computation of SC to be only approximately correct. The accuracy increases only when the bitstream is long enough to better approximate the probability. This significantly reduced the accuracy of SC based implementation.


$$\frac{\begin{array}{cccc} 6/8 & \text{11011101} & 6/8 & \text{10111011}\\ 4/8 & \text{10011001} & 4/8 & \text{01101010} \end{array}}{\begin{array}{cccc} 6/8 & \text{10011001} & 3/8 & \text{00101010} \end{array}}$$

To resolve this problem, prior work proposed deterministic Stochastic Computing as an algorithm that computes on deterministic bit streams. This version of stochastic computing enables reduced area, reduced latency, and produces completely accurate results (Jenson and Riedel, 2016). By properly structuring input bitstreams, completely accurate results can be produced with no random-fluctuation or correlation errors. For example, as shown below, by using 100 to represent $\frac{1}{3}$ and 1110 to represent $\frac{3}{4}$, then repeat these two small streams until it pairs every bit from one bitstream with every bit of the other bitstream exactly once, to get an accurate result of $\frac{3}{12}$:


$$\frac{\begin{array}{cc} 1/3 & \text{100100100100}\\ 3/4 & \text{111011101110} \end{array}}{\begin{array}{cc} 3/12 & \text{100000100100} \end{array}}$$

**Figure 1:**
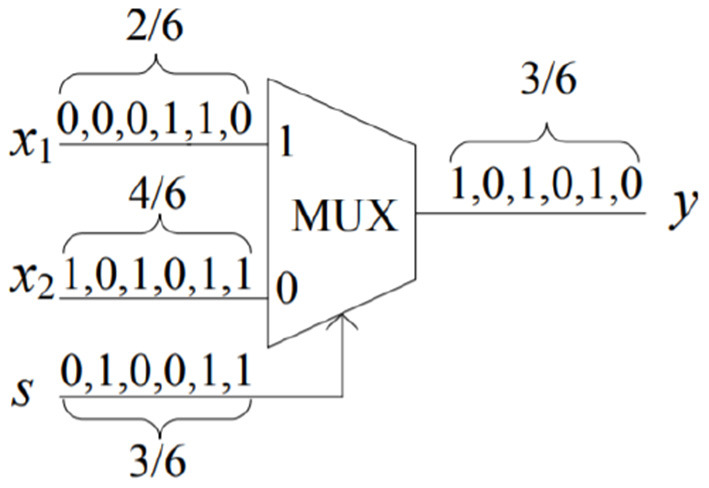
Stochastic implementation of addition.

Deterministic Stochastic Computing can also perform scaled addition and subtraction with a MUX operation. For example, as shown in Figure 1, we have a bitstream of 000110 representing $\frac{2}{6}$ as the first input, 101011 representing $\frac{4}{6}$ as the second input, and 010011 representing $\frac{3}{6}$ as the scale factor. By taking the MUX operation between these bitstreams, we get 101010 representing $\frac{3}{6}$, which is equal to ($\frac{3}{6}$)($\frac{2}{6}$) + (1 - $\frac{3}{6}$)($\frac{4}{6}$).

### 2.3. Processing in Memory

HD Computing is light-weight enough to run with acceptable performance on CPUs (Imani et al., 2019b). However, utilizing a parallel architecture can significantly speed up HD execution time. Imani et al. showed two orders of magnitude speed up when HD runs on GPU (Imani et al., 2017a). Salamat et al. proposed a framework that facilitates fast implementation of HD algorithms on FPGA (Salamat et al., 2019). Due to the bit-level operations in HD, which are more suitable for FPGAs than GPUs, they claimed up to 12× energy and 1.7× speed up over GPUs. HD requires much less memory than DNNs, but the required memory capacity is still beyond the local cache of many devices. Thus, an excessive amount of energy and time is spent moving data between these devices and their main memory (off-chip memory in the case of FPGAs). This problem is further exasperated with the use of stochastic bitstreams as we now need a long stream of bits (100*s*) for each value rather than a small amount such as 16−*bit* floating point.

To resolve this, prior work used PIM architectures, where processing occurs in memory, reducing the time and energy of data movement (Imani et al., 2019c, 2020; Li et al., 2020). In FELIX (Gupta et al., 2018), a *digital* PIM architecture was proposed. However, digital PIM operations are significantly slower than equivalent analog PIM operations. Digital PIM also suffers in performance with reduction operations. Prior work has also implemented HD Computing in an analog PIM ReRAM architecture to achieve faster execution times as in analog PIM, the accumulation is not a bottleneck (Morris et al., 2021). However, the bottleneck in analog PIM is the use of ADCs. ADCs are the highest energy overhead in the architecture (Shafiee et al., 2016). This is mitigated in prior work by reducing the ADC bitwidth and computing with inexact conversions (Morris et al., 2021). In this work, we eliminate the issue of ADCs with a hybrid approach between analog and digital PIM by removing the use of ADCs entirely.

## 3. HD with Stochastic Computing

**Figure 2:**
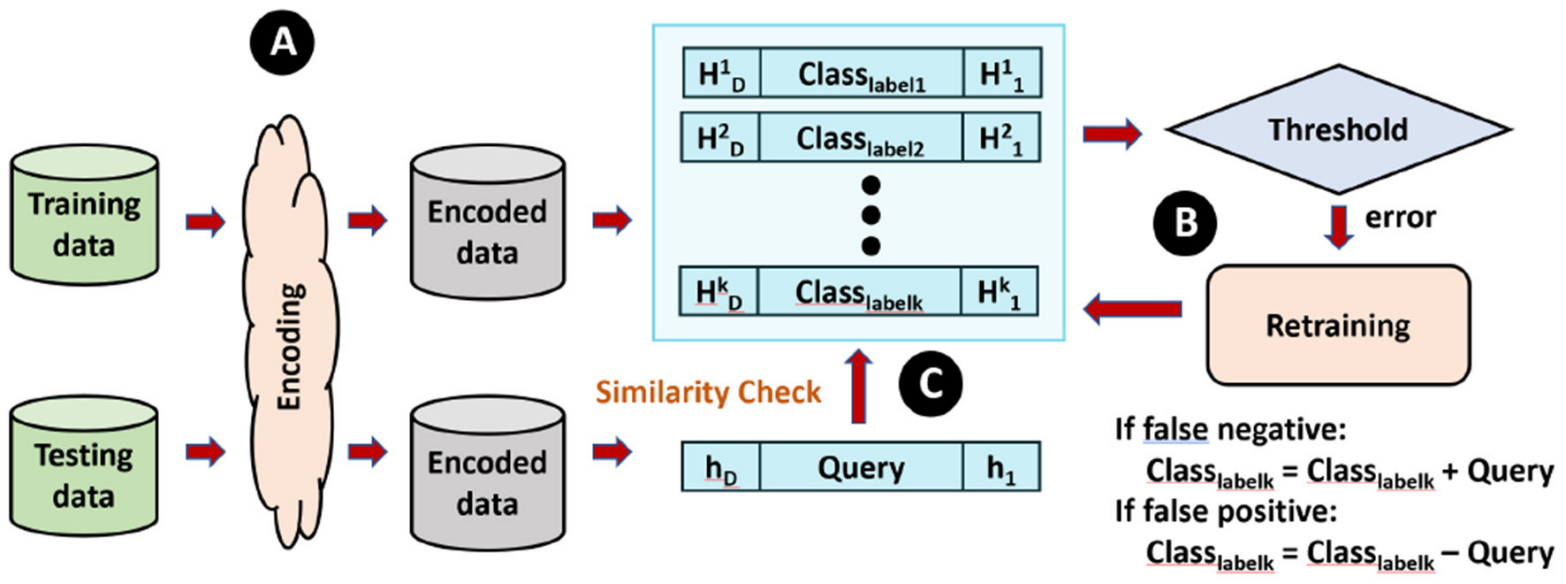
Overview of how baseline HD Classification is constructed and performs training and inference.

In this paper, we propose Stochastic-HD, a novel algorithm that takes the advantages of both HD computing and Stochastic computing. Although HD computing is more efficient than more traditional methods with its simple highly parallel operations. The main bottle-neck in highly parallel architectures such as PIM is the need for reduction operations such as accumulation at the end of a dot product. By mapping HD Classification and HD Clustering to the stochastic domain, we are able to perform all operations as highly parallel bitwise operations and remove all reduction operations. We first go over Stochastic-HD Classification that consists of three main modules shown in Figure 2: encoding, training, and testing.

### 3.1. Encoding

#### 3.1.1. Baseline HD

HD computing encoding maps each *n* dimensional feature vector to a *D* dimensional binary hypervector. We utilizes a random projection encoding presented in Imani et al. (2019f). The goal of encoding is to map a feature vector **F** = {*f*_1_, *f*_2_, …, *f*_*n*_}(*f*_*i*_∈ℕ) to a *D* (e.g., *D* = 10, 000) dimensional space vector: **H** = {*h*_1_, *h*_2_, …, *h*_*D*_}. The encoding first generates *D* dense bipolar vectors with the same dimensionality as original domain, **P** = {**p**_1_, **p**_2_, …, **p**_*D*_}, where ${\text{p}}_{i}\in {\left\{-1,1\right\}}^{n}$. Thus, to encode a feature vector into a hypervector, we perform a matrix vector multiplication between the projection matrix and the feature vector using **H** = *sign*(**PF**), where *sign* is a sign function which maps the result of the dot product to +1 or -1. However, this encoding process is very computationally expensive as it consists of thousands of arithmetic operations with a high dimension *D*. As a result, we want to apply SC as a light-weight hardware-friendly encoding algorithm to enable parallelism to solve this problem.

#### 3.1.2. Stochastic HD

Instead of using multiplication, we use logical AND and XOR to accomplish the same calculation. Since the projection matrix **P** = {**p**_1_, **p**_2_, …, **p**_*D*_} has ${\text{p}}_{i}\in {\left\{-1,1\right\}}^{n}$, to apply SC, we use a sign bit of 0 to represent a positive value and 1 to represent a negative value before normal stochastic bitstreams (Zhakatayev et al., 2018). We do the same for the feature vectors **F** = {*f*_1_, *f*_2_, …, *f*_*n*_}.

As for the stochastic bitstreams, as the example shows in Section 2.2, we generate the stochastic bitstreams first by looking for two fractions to represent the two values we want to multiply with, and then convert them into small bitstreams. As a result, we first cast the input data from float into int. Since in the original process, after matrix multiplication, the value would be cast into +1 or -1, casting the input data from float into int does not cause significant accuracy drop. In addition, since each multiplication of projection matrix and feature vector is composed of multiplication of +1/-1 with *f*_*i*_, to apply deterministic SC, we could directly use a stream of 1's, of length *max*(*abs*(|*f*_*i*_|)) to represent each element in projection matrix, and represent each feature *f*_*i*_ by using $\frac{f_{n}}{max\left(abs\left(f_{i}\right)\right)}$ and thus corresponding bitstream. Thus, with all values converted into bitstreams, we were able to take XOR for sign bit and AND for the rest bits to generate accurate results for the original multiplication calculation. After this, we count the number of 1's for both positive sign bits and negative sign bits and do subtraction. If the result is positive, we will cast the result into +1, and otherwise -1. With our implementation, all the multiplications and additions are converted into parallelizable AND or XOR operations, which is more efficient in hardware.

It is noteworthy that this conversion to the stochastic domain is only done once for the projection matrix and the same matrix can then be stored and reused for all encoding operations. Furthermore, for training, we only perform the conversion of the feature vector once as well because we store the encoded HVs. It is only during inference that we need to convert a feature vector to the stochastic domain online. Finally, for all of our conversions, we make use of a simple pulse generator because we are not generating random numbers, but deterministic ones (Li et al., 2016).

### 3.2. Initial Training

During initial training, the model is initialized through element-wise addition of all encoded hypervectors in each existing class. The result of training is *k* hypervectors each with *D* dimensions, where *k* is the number of classes. For example, the *i*^*th*^ class hypervector can be computed as: ${\text{C}}_{i}=\underset{\forall j\in class_{i}}{\sum }{\text{H}}_{j}$. In addition, to limit the data range of the class hypervectors for shorter stochastic bitstream length, we quantized the class hypervector uniformly. This makes sure that the range of the class hypervectors and the range of test hypervectors are not the same, which is important in similarity checking because we want to make sure that simple repetition of stochastic sequence is able to map each bit exactly once.

### 3.3. Similarity Check and Inference

#### 3.3.1. Baseline HD

**Figure 3:**
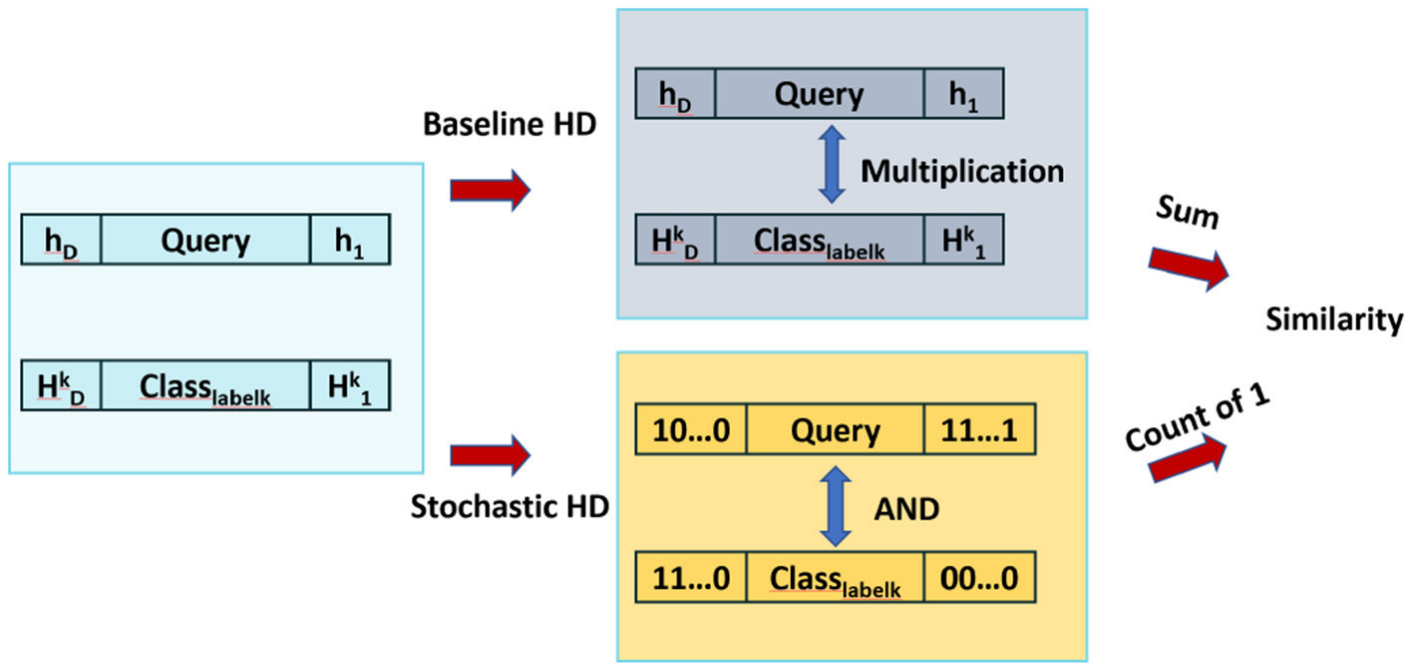
Comparison of similarity check of Baseline HD and Stochastic HD.

As shown in Figure 3, similarity checking is done by taking cosine similarity of each query and the class hypervector. However, this implementation requires thousands of multiplications and additions, which is significantly computationally expensive. As a result, to enable more hardware-level parallelism, we provide similarity checking with SC.

#### 3.3.2. Stochastic-HD

Figure 3 compares the similarity checking of Baseline HD Computing and Stochastic-HD. Instead of cosine similarity, Stochastic-HD uses deterministic SC to generate bitstreams for each data point **h_i_** in the Query ${\text{H}}_{\text{i}}^{\text{k}}$ and in the class hypervectors of the encoded model. Then, we put these two bitstreams through an AND gate to produce the result bitstream. To compare the similarity, we simply count the number of 1′*s* of each result bitstreams, and the one with most 1′*s* represents a larger value, thus closer similarity.

**Figure 4:**
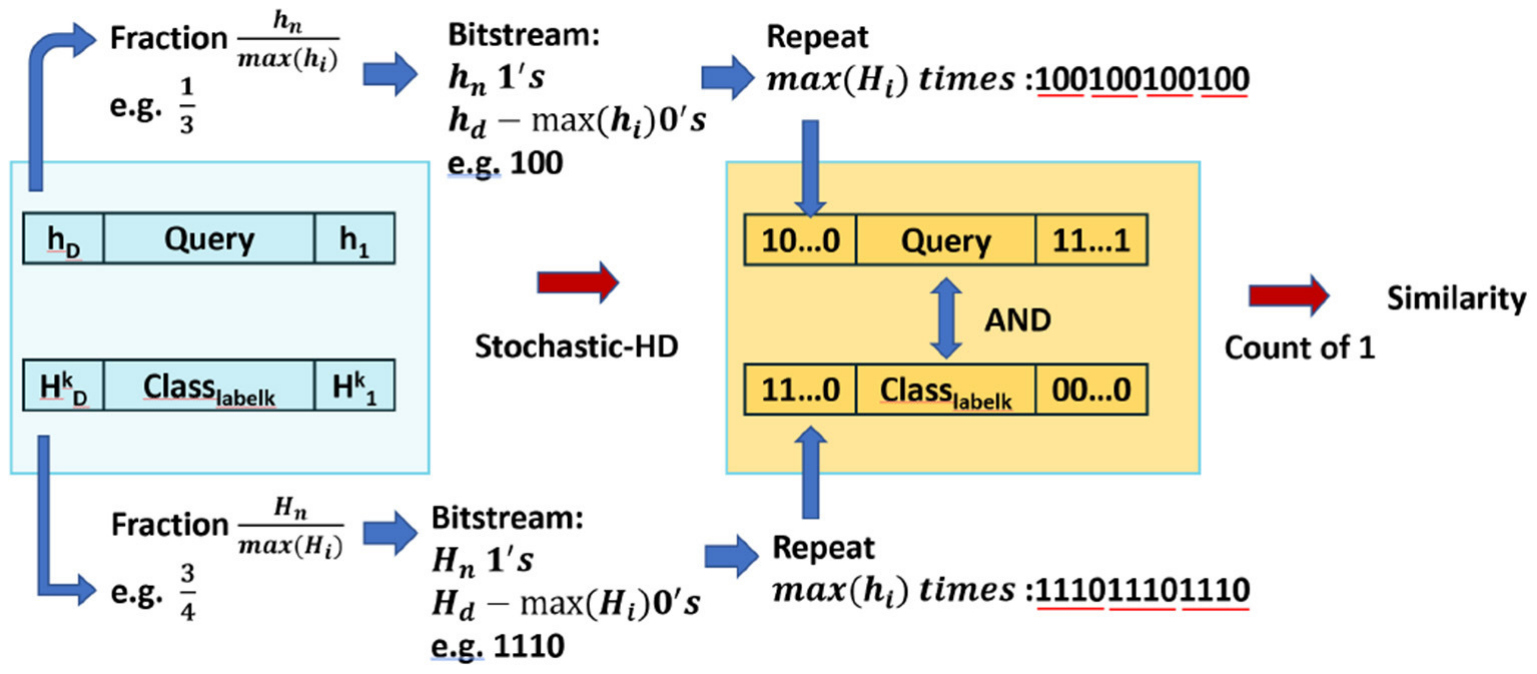
An overview of similarity checking is done with Stochastic-HD

As in Section 2.2, the way we generate the deterministic stochastic bitstreams is to first look for two fractions that represents the values from query and class hypervector, and then convert them into small bitstreams. Since we are able to get an integer encoding from Stochastic-HD encoding and initial training, we first make all the values in the encoded hypervector to be positive values. Then, the fraction is naturally generated by $\frac{h_{n}}{max\left(h_{i}\right)}$ and $\frac{H_{n}}{max\left(H_{i}\right)}$ for the n^th^ element of query and class hypervector as shown in Figure 4. This means that the bitstream length for query and class hypervector is *max*(*h*_*i*_) and *max*(*H*_*i*_), and the number of 1's in the bitstream is *h*_*n*_ and *H*_*n*_. Next, we repeat these small bitstreams to form two large two bitstreams until it pairs every bit from one bitstream with every bit of the other bitstream exactly once. This means that we repeat *max*(*H*_*i*_) and *max*(*h*_*i*_) times for each *h*_*i*_ and *H*_*i*_ respectively as shown in Figure 4.

Now with two bitstreams, we will input them into an AND gate and then count the number of 1′*s* to represent the similarity. For example, in Figure 4, to calculate the similarity between Query *h* and Class hypervector *H*^*k*^, suppose that $\frac{h_{n}}{max\left(h_{i}\right)}$ is $\frac{1}{3}$ and $\frac{H_{n}}{max\left(H_{i}\right)}$ is $\frac{3}{4}$, then the small bitstreams we generated for them is 100 and 1110. Then, repeat them until every bit in the first bitstream matches every bit in the other bitstream exactly once: 100100100100 (repeat *max*(*H*_*i*_) = 4 times) and 111011101110 (repeat *max*(*h*_*i*_) = 3 times). With all conversion done for all elements in two hypervectors, we will input these two bitstreams into an AND gate array. The number of 1's in the output represent the similarity. And the class with highest similarity would be our prediction.

By supporting both encoding and similarity check, Stochastic-HD can support inference. First, the new incoming sample is encoded into a stochastic hypervector using the same encoding used to train the initial model. This hypervector will be used as the query hypervector. Then, the query hypervector is input to the similarity checking phase and the most similar class hypervector is chosen as the output.

### 3.4. Retraining

#### 3.4.1. Baseline HD

Baseline HD performs retraining by first encoding the input sample into a HV, called the query HV. HD Computing then computes the similarity between operation between the query HV and the initially trained class HVs. As discussed, this operation is costly as it uses cosine similarity as the similarity metric, which limits parallelism with the accumulation. If the output class is correct, the HD model is not updated. However, if the output is incorrect, the model is updated by i) adding the incorrectly classified hypervector to the correct class (${\tilde{\text{C}}}_{correct}={\text{C}}_{correct}+\text{H}$), and (ii) subtracting it from the class to which it is wrongly matched (${\tilde{\text{C}}}_{wrong}={\text{C}}_{wrong}-\text{H}$). Performing this retraining operation over the entire training set for multiple epochs yields a more accurate model. One advantage of HD Computing is unlike other machine learning methods, the initial model is fairly accurate and HD Computing only needs a few iterations of retraining, rather than hundreds or thousands (Imani et al., 2017b).

#### 3.4.2. Stochastic-HD

Retraining in Stochastic-HD is very similar to baseline retraining, however, there are key differences. In Stochastic-HD, we replace the costly encoding step and similarity step with deterministic stochastic operations to increase parallelism. Again, if the output class is correct, our model does not need to be updated. If it is wrong, we need to update our stochastic bitstreams for the class HVs. Stochastic-HD needs to perform the same update as the baseline design, but on stochastic bitstreams instead of traditional number representations. To do this, we perform stochastic addition and subtraction. We cannot directly compute the addition or subtraction as it can result in values greater than 1. Instead we perform scaled addition and subtraction. This is implemented in deterministic stochastic computing with a simple MUX. As a result, the update equations for Stochastic-HD are as follows: i) adding the incorrectly classified hypervector to the correct class (${\tilde{\text{C}}}_{correct}=\text{s}{\text{C}}_{correct}+\left(1-\text{s}\right)\text{H}$), and (ii) subtracting it from the class to which it is wrongly matched (${\tilde{\text{C}}}_{wrong}=\text{s}{\text{C}}_{wrong}-\left(1-\text{s}\right)\text{H}$), where **s** is the scale factor. **s** Can be seen as a learning rate. With a larger scale factor the impact of retraining is less as more proportion is given to the class HV and less to the query. With a smaller scale factor the model is able to change more dramatically based on the query HVs value. We further explore this parameter in Section 5.

### 3.5. HD Clustering

#### 3.5.1. Baseline-HD

The first step of HD Clustering is to encode the data into high-dimensional space. HD Clustering then operates on the encoded HVs as the main datatype. HD Clustering initially selects random centers. It then iterates through all of the encoded data points while comparing them with the cluster centers using a similarity metric and assigning each point to the center it is most similar to. In K-means, that similarity metric is the Euclidean distance. In HD Clustering, we utilize cosine similarity for non-binary values, but Euclidean distance could also be used. However, HD maps data into high dimensional space, *D* = 10, 000, so calculating cosine similarity is much more efficient so this is what we evaluate in this paper. After all the points are labeled, the new centers are chosen and the process is repeated until convergence or the maximum number of iterations is reached. Convergence occurs when no point is assigned to a different cluster compared to the previous iteration.

#### 3.5.2. Stochastic-HD

The Stochastic version of HD Clustering has two key differences from the standard HD Clustering algorithm: (1) the exact encoding used by the state of the art HD Clustering algorithms is replaced with our Stochastic encoding from Section 3.1. (2) The similarity check is replaced with our Stochastic similarity from Section 3.3.

## 4. Stochastic-HD Hardware Design

In both encoding and similarity checking, there are vector-matrix multiplications (VMM) that involve dot products between the input vector and each column of the matrix. In this section, we detail our implementation of stochastic VMM in Stochastic-HD. We first propose an in-memory search-based binary vector matrix multiplication and then extend it to support input bitstreams in Stochastic-HD. We show how the same memory block design can be used for both Stochastic-HD encoding, similarity check, and retraining.

### 4.1. Search-Based Binary VMM

**Figure 5:**
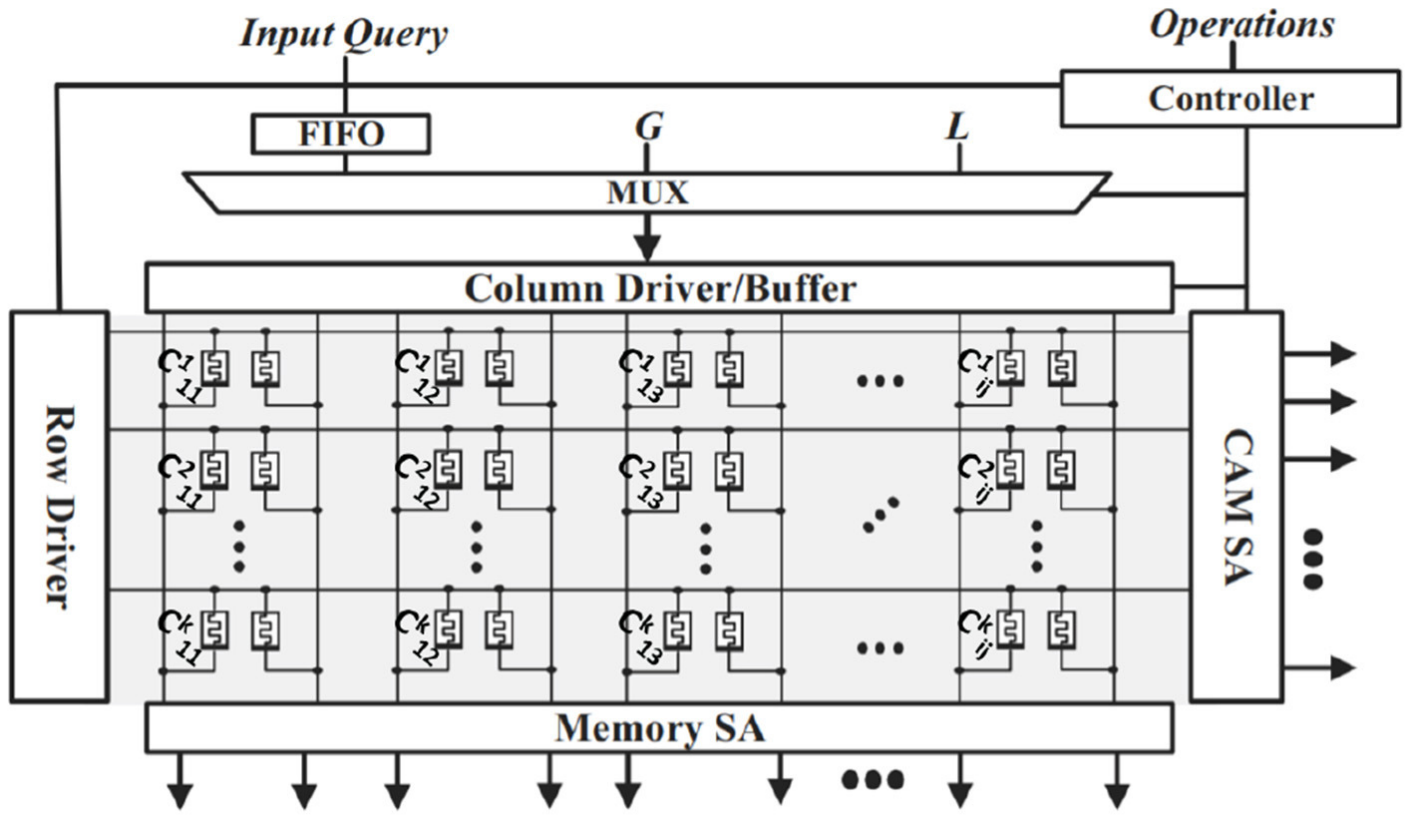
Stochastic-HD memory architecture overview.

AnalogPIM is based on the *magnitude* of accumulated current, while in-memory search is based on discharging speed of wordlines. We propose a hybrid approach that provides us with higher precision but without using DACs/ADCs. The memory layout of our implementation can be seen in Figure 5. In associative search, when the data stored in a memory row is the same as the input query, that wordline discharges the fastest (Imani et al., 2017c). Moreover, a match between “1” and “1” takes the same time to discharge the memory as a match between “0” and “0”. Also, the discharging is slower for a mismatch as compared to a match (Imani et al., 2017c). However, in our case, we need to implement an AND operation and differentiate the match operation between (1-1) from the rest of the three combinations (0-0, 0-1, 1-0).

In our design, we only apply “1”s at the input query, while leaving the lines corresponding to input “0” floating. This avoids the occurrence of 0-0. Floating the lines results in the current flowing through highly resistive path, which is similar to 1-0 and 0-1 mismatch cases. Although our floating input has a higher resistive path as compared to the mismatch current paths, both are the similar in magnitude as compared to the low resistive path of 1-1 match. Floating columns have traditionally posed issues with crossbar arrays, such as computation errors and limiting the scalability of the array. However, in our design, we do not need exact results for two reasons. First, HD Computing has been shown to be resilient to errors in computation (Morris et al., 2021). Second, during our inference operation, we do not need the exact sorted ordering of the similarities, we just need to know the top match. Furthermore, to solve scalability issues, we do not perform the matching along the entire crossbar, we perform the operation in chunks (Imani et al., 2016).

The discharging characteristic of a whole memory row represents the accumulative effect of all the AND operations between the input and stored hypervectors. Hence, the search output of each row represents a dot-product between the input query and the memory row. With more occurrences of “1-1” in the hypervectors, the faster the discharge, the higher the dot product value. This happens for all rows in parallel. The output of the entire memory block is a vector-matrix multiplication. We can increase the precision of the output dot product by sampling the output. Unlike AnalogPIM designs, we sample the output in the time domain. For example, to generate a dot product with 4 bits of precision, we use the same search circuits but latch the output at 16 different time instants (Gupta et al., 2022). Figure 5 has an example of a query search for a hypervector and how the hypervectors are laid out in memory. The class hypervectors are stored in the rows of the memory and shown by $C_{ij}^{k}$ where k is the class label, i is the dimension, and j is the stochastic bit. Clearly, the entire bitstream of a hypervector cannot fit in one row, so the hypervectors are spit up between multiple crossbars. For searching the input buffer distributes the query hypervector along the vertical bitlines. Then, the sense amplifier connected to the horizontal bitlines detects the highest match by sampling the discharge rate.

### 4.2. Encoding with Stochastic-HD Binary VMM

We perform search-based binary VMM n times for n-bit input bitstreams. In addition to the AND operation between stochastic bitstreams, we perform XOR operations between the input sign-bits and the stored projection matrix. To perform XOR, we activate the bitlines corresponding to both “0” and “1” for the sign-bit, unlike AND operation where we just activate “1”s. We use the accumulators at the memory periphery to add the dot product outputs of the n iterations. Since the final encoded hypervector consists of {-1, 1}, we take the sign bit of the accumulated output. Since each bit of stochastic bitstream has the same significance, we don't require shift-and-accumulate circuits used by traditional analogPIM designs and can utilize simple accumulators with low bit-precision. This operation reintroduces an accumulation into our design as a potential bottleneck. However, as this is during encoding, we only need to perform this operation once for training and retraining as we store the resulting encoded hypervectors for reuse. This operation is still used during inference to encode the query. However, because we can utilize simple accumulators with low precision, our resulting architecture is still 43× more energy efficient than the SoA PIM designs for HD Computing (Imani et al., 2019g).

### 4.3. Similarity Check with Stochastic-HD Binary VMM

During the similarity check, we need to take a dot product between an encoded hypervector with input elements {-1, 1} and a class hypervector with multi-bit elements. To implement this in Stochastic-HD PIM, we distribute a class hypervector over multiple memory rows. Each memory row stores 1 bit of the class bitstream for all dimensions. The dot product outputs of all rows corresponding to a class are added together to get the final dot product of the input with that class. The class with the highest dot product is selected as the output class.

### 4.4. Retraining with Stochastic-HD

As mentioned in Section 2, deterministic stochastic computing addition and subtraction is implemented with simple MUX blocks. Since in HD Computing we only need to modify two class HVs during each retraining sample, the correct class and the incorrect class. In our circuit, we perform each update to the correct class and incorrect class separately. We perform the AND part of the MUX operation by distributing the class hypervector over multiple memory rows and again only apply “1”s at the input query as the scale factor to perform the AND operation. We perform the same operation in a separate memory block for the AND operation between the query hypervector and the inverted scale factor. Our design then takes the OR operation between the output of both operations to finish the MUX operation. Subtraction is implemented by simply flipping the sign bit of the second operand.

### 4.5. Clustering with Stochastic-HD

As described in Section 3.5, for HD Clustering, instead of using Euclidean distance, we use cosine similarity to measure the distance between the samples and the cluster centers. This makes mapping HD Clustering onto Stochastic-HD simple. The similarity checking in HD Classification and HD Clustering is the same, therefore, we can use the same similarity checking accelerator block used for HD Classification to accelerate the similarity checking in HD Clustering. Additionally, we use the same encoding for Clustering and Classification, so that accelerator can be reused as well. Therefore, to map HD Clustering to Stochastic-HD, we input the samples in the original feature domain into our encoding block. Then, to update the distances between the samples and the cluster centers, we input the cluster centers into the inference accelerator as the class HVs and the samples as the query HVs. This then gives us both the distance in similarity between each sample and all the cluster centers as well as the cluster that each sample is most similar to. The next step of the HD Clustering algorithm, which is to chose the next cluster centers is too complex to accelerate in PIM. However, 98% of the time is spent on encoding and similarity checking. Therefore, offloading updating the cluster centers to the host CPU does not incur a significant amount of overhead.

## 5. Experimental Results

### 5.1. Experiment Setup

We implemented key components of Stochastic-HD at the circuit level to get accurate performance and energy consumption estimates. For this we simulate with Cadence Virtuoso with a 45 nm CMOS process technology. We use the VTEAM memristor model (Kvatinsky et al., 2015) for our memory design simulation with *R*_*ON*_ and *R*_*OFF*_ of 10*kΩ* and 10*MΩ* respectively. We leverage these models in cycle-accurate simulation of Stochastic-HD. Furthermore, to account for any process variability, we utilize Monte Carlo simulations with 10% process variability. The model and simulation parameters are also tuned to the WINBOND Rram chip (Ho et al., 2017).

We tested Stochastic-HD encoding, similarity checking, and retraining performance on CPU using Python. For comparison, we utilized a quantized version of HD computing as the baseline HD, which is also implemented in Python (Imani et al., 2019a). We use *D* = 2048 as the default dimensionality across all tests except when varying the dimensionality. We use an Intel i7 7600 CPU with 16GB memory for our baseline CPU. We use performance counters to measure CPU power and execution time.

For Naive-PIM, we implement HD Classification and Clustering using the digital processing in memory technology proposed in Gupta et al. (2018).

We tested our proposed classification approach on six applications: Speech Recognition (ISOLET)^1^, Face Detection (FACE) (Griffin et al., 2007), Activity Recognition (UCIHAR)^2^ (PAMAP2)^3^. Gesture recognition(EMG) (Benatti et al., 2014), Cardiotocography (CARDIO)^4^. For clustering, we tested on six datasets from the Fundamental Clustering Problem Suite (Ultsch, 2005). All dataset details are shown in Table 2.

**Table 2:** Dataset information.

**Dataset**	**Type**	**# Classes**	**# Train data**	**# Test data**	**# Features**
UCIHAR^2^	Classification	6	6,213	1,554	561
CARDIO^4^	Classification	2	1,913	213	21
FACE (Griffin et al., 2007)	Classification	2	22,441	2,494	608
ISOLET^1^	Classification and clustering	26	6,238	1,559	617
Hepta (Ultsch, 2005)	Clustering	7	N/A	212	3
Tetra (Ultsch, 2005)	Clustering	4	N/A	400	3
Two diamonds (Ultsch, 2005)	Clustering	2	N/A	800	2
Wingnut (Ultsch, 2005)	Clustering	2	N/A	1,016	2
Iris (Ultsch, 2005)	Clustering	3	N/A	135	3

### 5.2. Stochastic-HD Scale Factor

**Figure 6:**
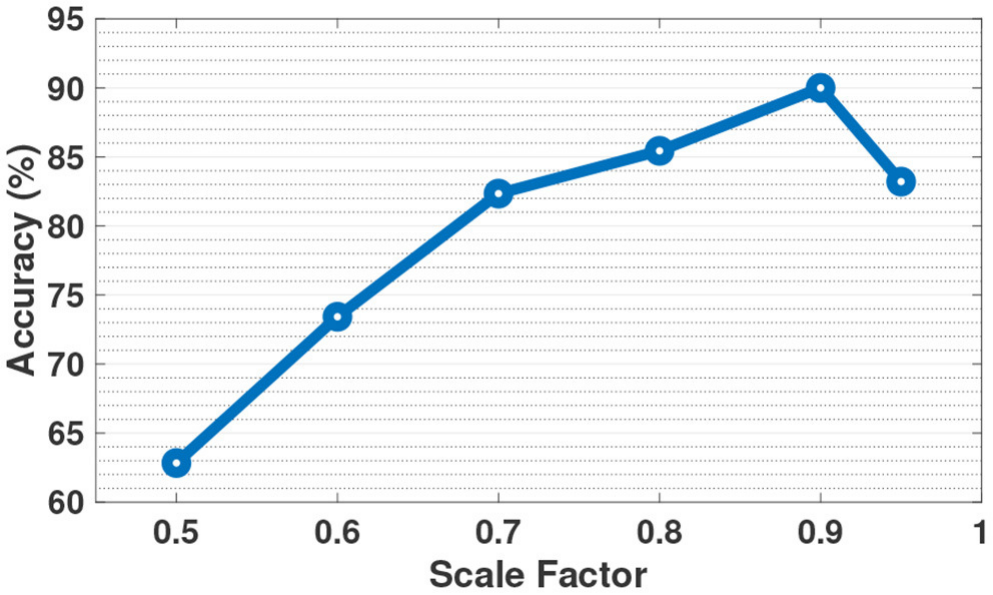
Impact of scale factor on the accuracy of Stochastic-HD on the ISOLET dataset.

Figure 6 shows the impact of the scale factor in our scaled addition during retraining on the accuracy of Stochastic-HD. As stated in Section 3.4, deterministic stochastic computing does not support simple addition and subtraction. Instead it is implemented as scaled addition and subtraction. Therefore, in Stochastic-HD, we have a scale factor parameter to tune. As the figure shows, with too low of a scale factor, such as 0.5, the class hypervectors are modified too heavily by the query. It is clear from the figure, that the class hypervector needs to be weighted more heavily than the query used to retrain the class hypervector. However, at the other end of the curve, if the class hypervector is weighted too much, the model does not change enough and does not increase in accuracy. As the curve shows, a scale factor of 0.9 gave the best results on the ISOLET dataset. This pattern remained true for all our tested datasets and we use a scale factor of 0.9 for all of our tests.

### 5.3. Stochastic-HD Classification Accuracy Comparison

**Figure 7:**
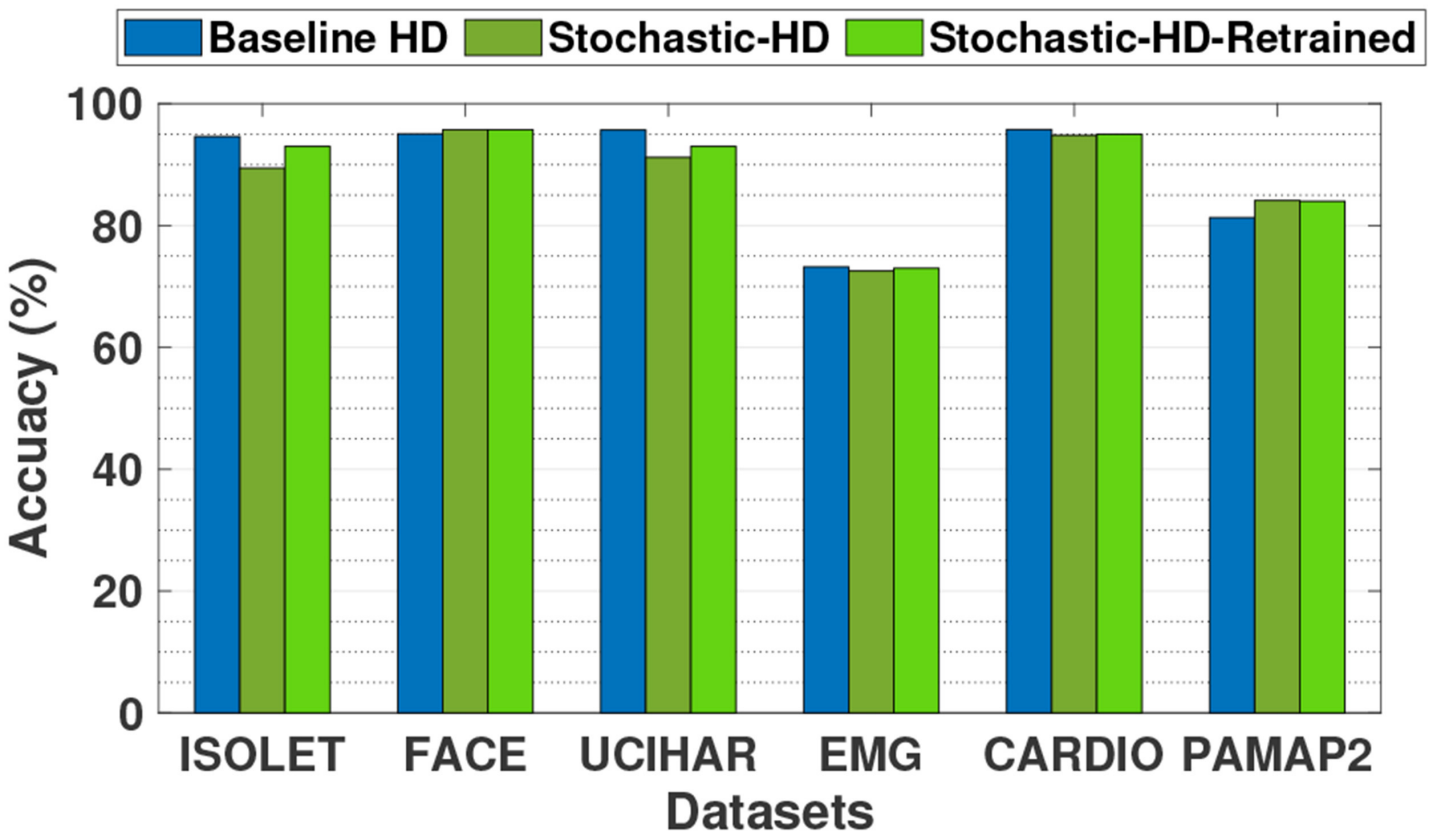
Classification accuracy comparison of Stochastic-HD with a CPU implementation (exact computation).

Figure 7 compares the classification accuracy of the baseline HD Computing, which uses exact operations with Stochastic-HD, which uses SC operations that are approximate. We also compare with Stochastic-HD supporting retraining. As the figure shows, Stochastic-HD is comparable in accuracy to baseline HD. It is noteworthy that our design using deterministic stochastic operations does not achieve the same accuracy as the baseline. This is because, although deterministic operations can have a one to one matching with conventional operations, it requires that the stochastic bitstream length be sufficient. In some cases, our bitstreams do not reach that requirement and result in some inaccurate computations. For the datasets ISOLET and UCIHAR, Stochastic-HD is slightly less accurate, losing 5% and 7% in accuracy respectively. These two datasets have more classes that have less separation than the other four datasets. This leads them to be more sensitive to approximate methods like SC, causing Stochastic-HD to be less accurate on them. However, for the other four datasets, Stochastic-HD is able to achieve the same accuracy. Furthermore, with the additional support of integrated retraining for Stochastic-HD, we are able to close the gap between the exact baseline HD and Stochastic-HD. Overall, compared to a baseline with exact computations, Stochastic-HD loses just 1.3% in accuracy on average and Stochastic-HD with retraining loses just 0.3% in accuracy on average.

Table 3 demonstrates the impact on the dimensionality of the HD model on accuracy for both the Baseline-HD implementation and Stochastic-HD on ISOLET dataset. As the table shows, the accuracy very slightly increases as the dimensionality is also increased for both the Baseline-HD and Stochastic-HD. However, the more interesting comparison is with the Baseline-HD with a dimensionality of 1000 vs Stochastic-HD at 5000 dimensions. The Baseline-HD model is able to achieve the same accuracy as Stochastic-HD at this dimensionality and performance scales with dimensionality. However, this would only lead to a 15.9× speedup and 441, 889× energy efficiency gain with a naive implementation of the Baseline-HD in PIM. However, with Stochastic-HD we are able to achieve 174× speedup and 3, 588, 126× energy efficiency gain by mapping all of our inference operations to the stochastic domain. Thus, every operation is a simple bitwise computation that is easily implemented in PIM as well as parallelized. This is in contrast to the much more complex operations needed for a naive implementation of the Baseline-HD such as the element-wise multiplications and accumulations.

**Table 3:** Impact of dimensionality on accuracy of Stochastic-HD and baseline-HD of ISOLET dataset.

**Dimension**	**1,000**	**3,000**	**5,000**	**7,000**	**9,000**
Baseline-HD	89.8%	93.8%	94.0%	94.4%	94.2%
Stochasitc-HD	77.9%	88.1%	89.4%	88.7%	91.2%

### 5.4. Stochastic-HD Clustering Accuracy Comparison

**Figure 8:**
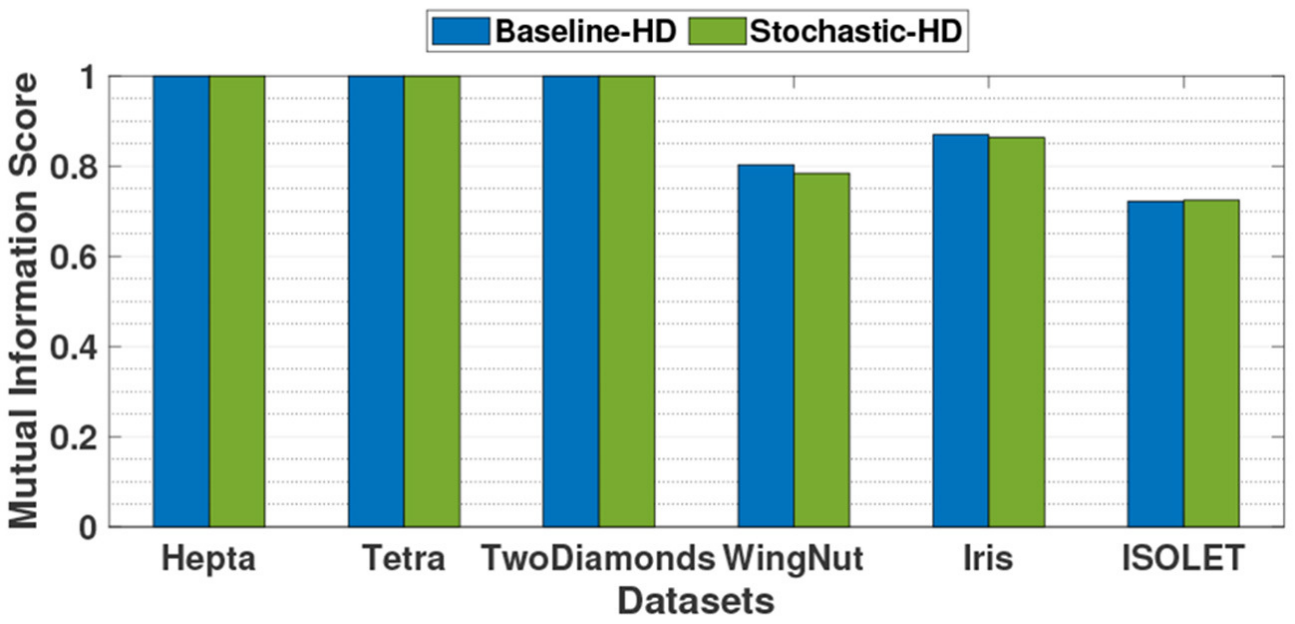
Clustering accuracy comparison of Stochastic-HD with a CPU implementation (exact computation).

Figure 8 compares the clustering accuracy of baseline HD with exact operations to Stochastic-HD clustering. We report mutual information score as the scoring metric for clustering. As the figure shows, Stochastic-HD is able to achieve comparable accuracy on all of the tested datasets. Overall, the average difference in mutual information score between baseline HD clustering and Stochastic-HD clustering is just 0.004 and the highest difference is 0.02.

### 5.5. Stochastic-HD vs. State-of-the-Art Accelerators

#### 5.5.1. Inference

**Figure 9:**
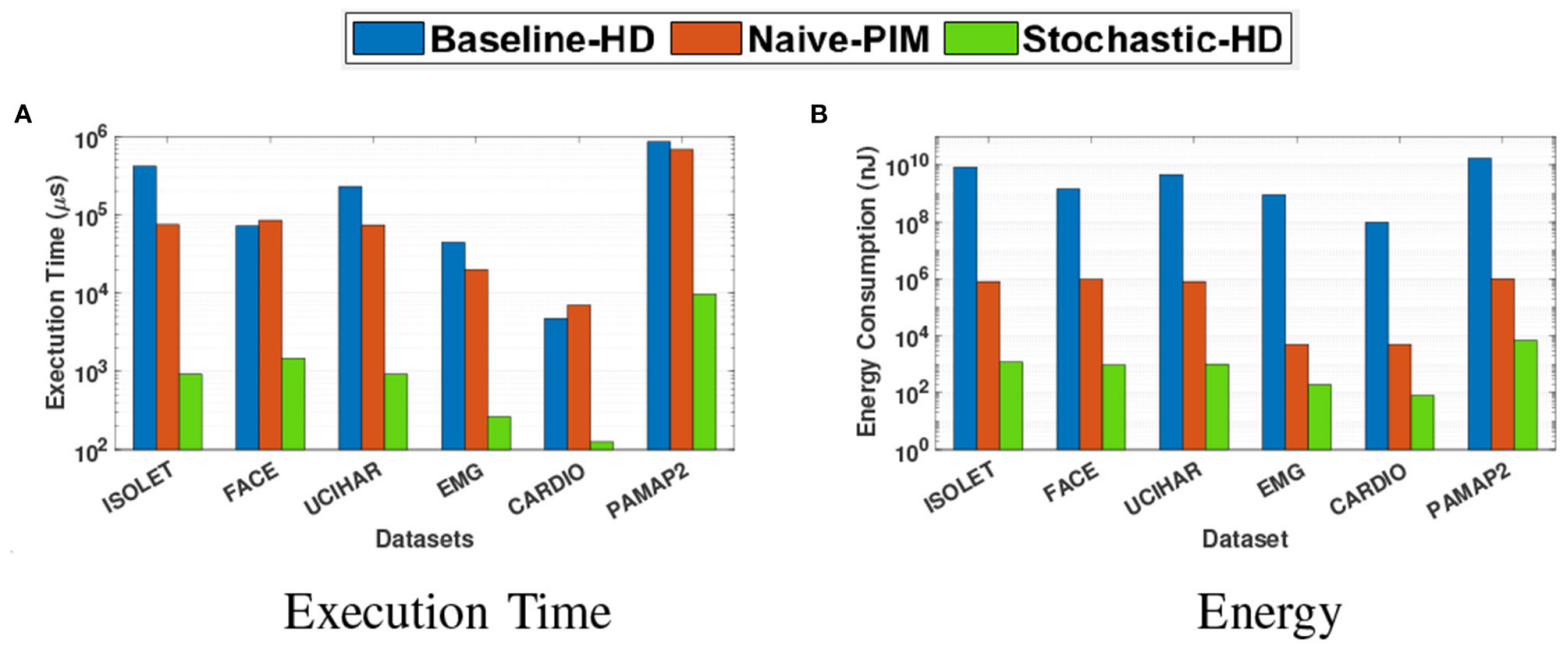
Comparison of the inference execution time and energy consumption of Stochastic-HD with CPU and Naive-PIM (Imani et al., 2019f,g). **(A)** Execution time. **(B)** Energy.

Figure 9 compares the execution time and the energy consumption of Stochastic-HD with a CPU implementation of the Baseline-HD as well as a Naive PIM implementation during inference. Naive-PIM simply maps the necessary operations for inference described in the state-of-the-art HD implementation in Imani et al. (2019f) onto the memory architecture as described in the state-of-the-art PIM work in Gupta et al. (2018). However, as noted in Section 1, simply implementing the Baseline-HD model into PIM leads to the element-wise multiplication and subsequent accumulation to bottleneck the parallelism and therefore performance. By applying SC in Stochastic-HD we are able to use simple bitwise operations throughout the inference process. With Stochastic-HD we achieve 52.8× speedup and 378× energy efficiency gain over Naive-PIM. However, Naive-PIM (Gupta et al., 2018; Imani et al., 2019f) can achieve the same accuracy as the software implementation.

#### 5.5.2. End-to-End

**Figure 10:**
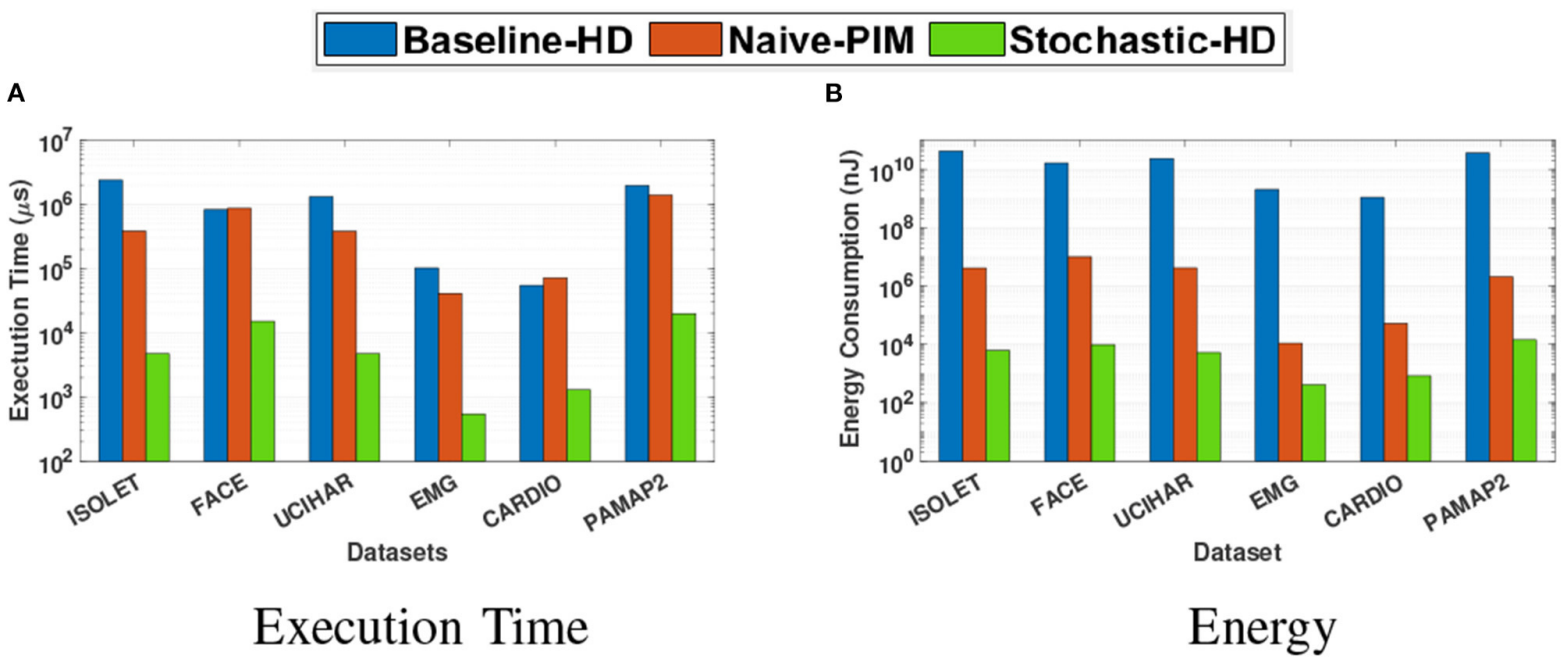
Comparison of the end-to-end execution time and energy consumption of Stochastic-HD with CPU and Naive-PIM (Imani et al., 2019f,g). **(A)** Execution time. **(B)** Energy.

We also compare the execution time and the energy consumption of Stochastic-HD with a CPU implementation of the Baseline-HD as well as a Naive PIM implementation for an end-to-end HD Computing solution in Figure 10. This includes encoding, training, retraining, and inference, whereas the previous figure only compares inference, which includes encoding the query and performing the similarity check. With Stochastic-HD with integrated retraining, we achieve 70× speedup and 456× energy efficiency gain over Naive-PIM, while closing the gap in accuracy further.

#### 5.5.3. Clustering

**Figure 11:**
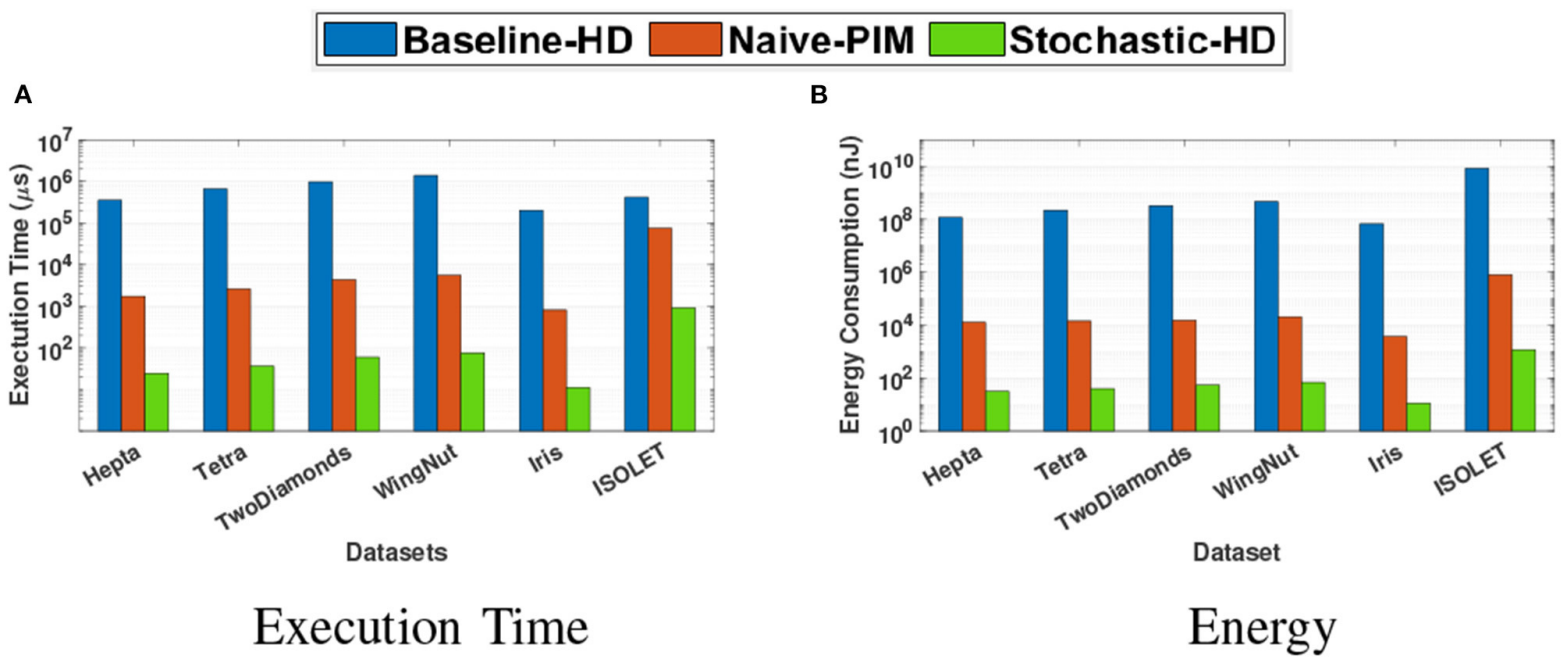
Comparison of the clustering execution time and energy consumption of Stochastic-HD with CPU and Naive-PIM (Imani et al., 2019f,g). **(A)** Execution time. **(B)** Energy.

We compare the execution time and the energy consumption of Stochastic-HD Clustering with a CPU implementation of the Baseline-HD clustering as well as a Naive PIM implementation of HD Clustering in Figure 11. This comparison shows the time it takes to encode and cluster all of the datapoints for one clustering iteration. With Stochastic-HD, we achieve 74× speedup and 392× energy efficiency gain over Naive-PIM.

#### 5.5.4. State-Of-the-Art

We compared Stochastic-HD with two state-of-the-art HD-PIM implementations in Datta et al. (2019) and SearcHD (Imani et al., 2019g). Stochastic-HD provides 13% higher accuracy on average than the (Datta et al., 2019) and 4.4% higher accuracy than SearcHD. Our results show that Datta et al. (2019) is 6.1× faster and SearcHD is 2.6× faster than Stochastic-HD. However, Stochastic-HD can achieve 10,618× energy efficiency over the design in Datta et al. (2019) and 43.1× energy efficiency over SearcHD. The higher energy efficiency of Stochastic-HD is due to the simpler bitwise stochastic operations. As compared to the best PIM design for HD (Imani et al., 2019g), Stochastic-HD is 4.4% more accurate and 43.1× more energy-efficient. Slightly worse performance of Stochastic-HD is the result of the Stochastic-HD's area-efficient approach as it uses the minimal memory required. Our design can however be extended to a bigger chip to achieve better performance. For example, a Stochastic-HD chip with 14× (174× ) larger area provides 11× (134× ) faster inference than the area-efficient Stochastic-HD, while consuming similar energy. This is due to the stochastic nature of our computations that can be parallelized. In contrast, the designs in Datta et al. (2019); Imani et al. (2019g) are limited to the parallelism provided by HD computing and incur the latency of multi-bit computations. As a result, the bigger Stochastic-HD chips are both faster and more accurate than the existing designs (Datta et al., 2019; Imani et al., 2019g).

### 5.6. Memory Overhead

While the encoding module in Stochastic-HD uses the same memory area as the traditional implementations, the memory area consumed by the similarity checking module and the retraining module increases with the bitstream length. In our evaluation, Stochastic-HD's similarity checking and retraining consumes 6× larger memory area as compared to the similarity check in baseline PIM designs. However, since encoding module consumes 66% of the total area, the similarity checking module consumes 5% of the total area, the retraining module consumes 3% of the total area, and the last 26% is consumed by memory peripherals such as the controller and SA, the total memory area overhead of Stochastic-HD is 40%.

## 6. Conclusion

In this paper, we implement Stochastic Computing(SC) end-to-end in HD Computing Classification and Clustering. We propose, Stochastic-HD, which combines both HD Computing and SC to perform classification tasks in PIM with highly parallel operations. We use SC because a naive implementation of existing HD work in digital PIM results in a bottleneck when we need element-wise multiplications and subsequent accumulations for the dot product operation. With Stochastic-HD, we represent each element of the query hypervectors with bitstreams, and replace the bottleneck operations with simple bitwise operations. With Stochastic-HD, we were able to reach a comparable accuracy with the Baseline-HD losing just 0.3% in accuracy while achieving 70× speedup and 456× energy efficiency gain over Naive-PIM (Gupta et al., 2018; Imani et al., 2019f) during classification. Furthermore, we achieve the same accuracy during clustering while achieving a 74× speedup and 392× energy efficiency gain over Naive-PIM. As compared to the best PIM design for HD (Imani et al., 2019g), Stochastic-HD is also 4.4% more accurate and 43.1× more energy-efficient.

## Data Availability Statement

Publicly available datasets were analyzed in this study. This data can be found here: https://archive.ics.uci.edu/ml/datasets/Daily+and+Sports+Activities, https://archive.ics.uci.edu/ml/datasets/cardiotocography, (Griffin et al., 2007), http://archive.ics.uci.edu/ml/datasets/ISOLET, (Benatti et al., 2014), https://archive.ics.uci.edu/ml/datasets/pamap2+physical+activity+monitoring, and (Ultsch, 2005).

## Author Contributions

JM: manuscript writing, figures, and experimental results. YH: experimental simulations and manuscript writing. SG and BK: hardware design. BA and TR: advising. All authors contributed to the article and approved the submitted version.

## Funding

This work was supported in part by CRISP, one of six centers in JUMP (an SRC program sponsored by DARPA), SRC Global Research Collaboration (GRC) grant, and NSF grants #1911095, #2003279, #2003277, #2100237, and #2120019.

## Conflict of Interest

SG was employed by the company IBM research. The remaining authors declare that the research was conducted in the absence of any commercial or financial relationships that could be construed as a potential conflict of interest.

## Publisher's Note

All claims expressed in this article are solely those of the authors and do not necessarily represent those of their affiliated organizations, or those of the publisher, the editors and the reviewers. Any product that may be evaluated in this article, or claim that may be made by its manufacturer, is not guaranteed or endorsed by the publisher.

## Footnotes

^1^UCI Machine Learning Repository: Isolet Data Set. Available online at: http://archive.ics.uci.edu/ml/datasets/ISOLET.

^2^UCI Learning Repository: Daily and Sports Activities Data Set. Available online at: https://archive.ics.uci.edu/ml/datasets/Daily+and+Sports+Activities. https://archive.ics.uci.edu/ml/datasets/Daily+and+Sports+Activities.

^3^UCI Machine Learning Repository: Pamap2 Physical Activity Monitoring Data Set: Available online at: https://archive.ics.uci.edu/ml/datasets/pamap2+physical+activity+monitoring.

^4^UCI Machine Learning Repository: Cardiotocography Data Set. Available online at: https://archive.ics.uci.edu/ml/datasets/cardiotocography.
